# I–II Loop Structural Determinants in the Gating and Surface Expression of Low Voltage-Activated Calcium Channels

**DOI:** 10.1371/journal.pone.0002976

**Published:** 2008-08-20

**Authors:** Joel P. Baumgart, Iuliia Vitko, Isabelle Bidaud, Artem Kondratskyi, Philippe Lory, Edward Perez-Reyes

**Affiliations:** 1 Department of Pharmacology, University of Virginia, Charlottesville, Virginia, United States of America; 2 Neuroscience Graduate Program, University of Virginia, Charlottesville, Virginia, United States of America; 3 CNRS, UMR 5203, Institut de Génomique fonctionnelle, Montpellier, France; 4 INSERM, U661, Montpellier, France; 5 Université Montpellier, 1 et 2, Montpellier, France; 6 Department of General Physiology, Bogomoletz Institute of Physiology, Kiev, Ukraine; University of Cincinnati, United States of America

## Abstract

The intracellular loops that interlink the four transmembrane domains of Ca^2+^- and Na^+^-channels (Ca_v_, Na_v_) have critical roles in numerous forms of channel regulation. In particular, the intracellular loop that joins repeats I and II (I–II loop) in high voltage-activated (HVA) Ca^2+^ channels possesses the binding site for Ca_v_β subunits and plays significant roles in channel function, including trafficking the α_1_ subunits of HVA channels to the plasma membrane and channel gating. Although there is considerable divergence in the primary sequence of the I–II loop of Ca_v_1/Ca_v_2 HVA channels and Ca_v_3 LVA/T-type channels, evidence for a regulatory role of the I–II loop in T-channel function has recently emerged for Ca_v_3.2 channels. In order to provide a comprehensive view of the role this intracellular region may play in the gating and surface expression in Ca_v_3 channels, we have performed a structure-function analysis of the I–II loop in Ca_v_3.1 and Ca_v_3.3 channels using selective deletion mutants. Here we show the first 60 amino acids of the loop (post IS6) are involved in Ca_v_3.1 and Ca_v_3.3 channel gating and kinetics, which establishes a conserved property of this locus for all Ca_v_3 channels. In contrast to findings in Ca_v_3.2, deletion of the central region of the I–II loop in Ca_v_3.1 and Ca_v_3.3 yielded a modest increase (+30%) and a reduction (−30%) in current density and surface expression, respectively. These experiments enrich our understanding of the structural determinants involved in Ca_v_3 function by highlighting the unique role played by the intracellular I–II loop in Ca_v_3.2 channel trafficking, and illustrating the prominent role of the gating brake in setting the slow and distinctive slow activation kinetics of Ca_v_3.3.

## Introduction

All excitable cells express voltage-gated Ca^2+^ channels, and in fact many such cells express a number of voltage-gated Ca^2+^ channel subtypes [1]. These channels offer a regulated entrance for extracellular Ca^2+^ into the cell interior, which can initiate a multitude of signaling cascades as Ca^2+^ itself functions as a second-messenger. In addition, the influx of positively charged Ca^2+^ ions can effectively depolarize the plasma membrane, which in turn can activate other voltage-gated ion channels. Ca^2+^ influx through voltage-gated Ca^2+^ channels has been shown to play important roles in diverse processes such as neuronal firing, neurotransmitter release and gene expression [2].

Despite rigorous investigation, little is known about the structural determinants of T-channels that underlie their activation at lower voltages than other voltage-gated channels, or what factors control their trafficking to the plasma membrane. Recent studies on the intracellular loop connecting repeats I and II (I–II loop) of Ca_v_3.2 indicate that it controls both the gating of the channel and its surface expression [3], [4]. The first 55 amino acids after the end of repeat I (proximal I–II loop) were found to contain a “gating brake,” the disruption of which led to channels that activated and inactivated at more hyperpolarized potentials. Deletions in the distal I–II loop primarily affected trafficking to the plasma membrane [3], and single nucleotide polymorphisms within this loop in Childhood Absence Epilepsy patients (CAE SNPs) bore significant effects on both Ca_v_3.2 channel biophysics and trafficking [3], [5]. Interestingly, the I–II loop in the α1 subunit of high voltage-activated (HVA) Ca^2+^-channels contains the binding site for β auxiliary subunits (Ca_v_β) that regulate the voltage dependence of activation, inactivation, probability of opening, pharmacology, and expression at the plasma membrane [6]. While T-type channels are believed to not complex with auxiliary subunits, given the robust effects that disruption of the α1-β interaction confers on HVA expression, it is interesting to consider potential roles for the I–II loop across all voltage-gated Ca^2+^ channels.

**Figure 1:**
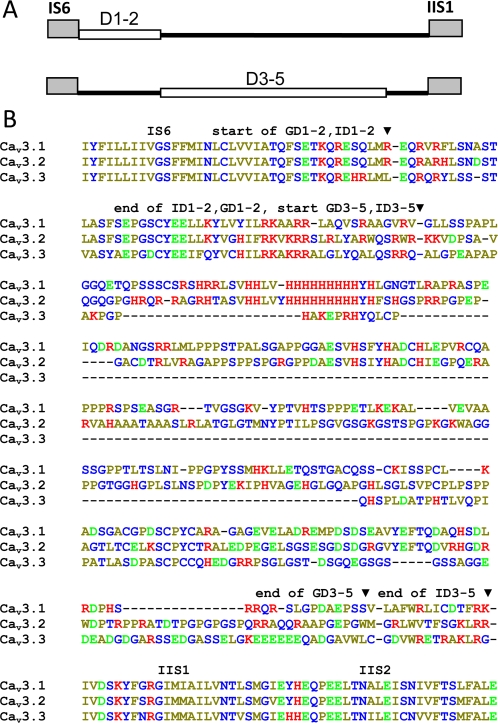
Location of deletions in the I–II loop. (*A*), Schematic representation of the I–II loop connecting repeat IS6 to repeat IIS1 in Ca_v_3.1 and Ca_v_3.3. Deleted regions are shown as open boxes. (*B*), Alignment of the I–II loop of human Ca_v_3 channels. Arrows indicate where deletions begin and end. Dashes represent gaps in the alignment. Amino acids are color-coded by their physical properties as follows: red, positively-charged; green, negatively-charged; blue, polar; and yellow, hydrophobic.

The biophysics of Ca_v_3.3 channels differ significantly from Ca_v_3.1 and Ca_v_3.2, with much slower kinetics and a more depolarized voltage-dependence, which endows Ca_v_3.3 with the ability to generate prodigious bursts of action potentials [7], [8], such as those observed in neurons from the reticular nucleus of the thalamus [9]. Accordingly, the structural determinants of Ca_v_3.3 gating have been the subject of much study, with two notable findings using chimeric analysis between Ca_v_3.3 and Ca_v_3.1 [10], [11]. One study concluded there was no single determinant, as swapping any single repeat produced modest effects [10]. A second study showed that moving repeat IV of Ca_v_3.3 into Ca_v_3.1 produced a chimera with slower inactivation kinetics, however, the reverse chimera, repeat IV of Ca_v_3.1 into Ca_v_3.3, did little to Ca_v_3.3 kinetics [11]. Regions involved in Ca_v_3.1 inactivation have been mapped to IIIS6 [12], IS6 [13], the selectivity filter [14], and the proximal C-terminus [15], which collectively suggests that Ca_v_3 channels resemble HVA Ca^2+^-channels, in that many regions are involved in determining their apparent rates of inactivation [16]. Sequence alignments [17] reveal that the proximal I–II loop is relatively well conserved across all three Ca_v_3 channels (Figure 1). In contrast, the distal portion of the I–II loop is less conserved, particularly for Ca_v_3.3, in which the I–II loop is considerably shorter than those of its Ca_v_3 counterparts. Based on the conservation of sequence, we hypothesized that the gating brake might be conserved in all Ca_v_3 channels. To further investigate the important role the I–II loop plays in the gating and surface expression of T-type channels, we performed a structure-function analysis of this intracellular region in Ca_v_3.1 and Ca_v_3.3 using selective deletion mutants. Specifically, we probed for the presence of both the gating brake with deletions of the proximal I–II loop, and for the expression brake in the distal I–II loop. Our data suggest the proximal region of the I–II loop serves as a gating particle in all T-type channels, while the medial/distal portion of the loop underlies varying effects on channel expression.

## Materials and Methods

### Site-directed mutagenesis

Fragments of human Ca_v_3.1 and Ca_v_3.3 cDNA were mutated using the QuikChange protocol (Stratagene, La Jolla, CA). To facilitate ligation of the full-length clone, a silent mutation was introduced that added a *Sal*I site into the Ca_v_3.1 cDNA, thereby allowing movements of D1–2 and D3–5 deletion as *Sal*I/*Sal*I fragments. The deletion mutants of Ca_v_3.3 were moved using *Sbf*I and *Avr*II restriction enzyme sites. The sequence of the mutated fragments were verified for each mutant by automated sequencing at the University of Virginia Biomolecular Research Facility. Following the strategy of Dubel et al. [18], the HA epitope and flanking linker (QEHYPYDVDPDYAVTFVD) was introduced into both Ca_v_3s at the extracellular loop connecting IS5 to the pore. The amino acids deleted in each of the constructs were as follows: Ca_v_3.1 (Genbank accession number AAF35287) GD1–2, 411–462; GD3–5, 468–722; Ca_v_3.3 (Genbank accession number AAM67414) ID1–2, 414–457; ID3–5, 470–587.

### Transfections

Human embryonic kidney 293 cells (HEK-293, CRL-1573, American Type Culture Collection, Manassas, VA) were grown in Dulbecco's modified medium F12 (DMEM/F-12, Invitrogen), supplemented with 10% fetal calf serum, penicillin G (100 units/ml), and streptomycin (0.1 mg/ml). Cells were transiently transfected with wild-type or deletion mutants of Ca_v_3.1 or Ca_v_3.3 channels using JET-PEI (PolyPlus, Illkirch, France). Cells for electrophysiology experiments were co-transfected with EGFP-expressing vector to allow for identification of expressing cells. Twenty-four to thirty-six hours post-transfection, the cells were dissociated by digestion with 0.25% trypsin plus 1 mM EDTA for two minutes, diluted 20-fold with DMEM, and then plated on glass cover slips. The cells were incubated at least four hours and up to two days prior to electrophysiological studies. The results were obtained from multiple transfections. Each construct was tested in at least two transfections, and control data were collected from a number of transfections.

### Electrophysiology

Whole cell Ca^2+^-currents were recorded using the following external solution (in mM): 5 CaCl_2_, 166 tetraethyl ammonium (TEA) chloride, and 10 HEPES, pH adjusted to 7.4 with TEA-OH. The internal pipette solution contained the following (in mM): 125 CsCl, 10 EGTA, 2 CaCl_2_, 1 MgCl_2_, 4 Mg-ATP, 0.3 Na_3_GTP, and 10 HEPES, pH adjusted to 7.2 with CsOH. Currents were recording using an Axopatch 200B amplifier, computer (Dell, Round Rock, TX), Digidata 1322 A/D converter, and pCLAMP 9.0 software (Axon Instruments, Union City, CA). Unless otherwise noted, data were filtered at 2 kHz and digitized at 5 kHz. Recording pipettes were made from TW-150-6 capillary tubing (World Precision Instruments, Inc., Sarasota, FL), using a Model P-97 Flaming-Brown pipette puller (Sutter Instrument Co., Novato, CA). Once filled with the internal solution the pipette resistance was typically 1.5–2.5 MΩ. Only recordings with minimal leak currents were analyzed (<100 pA), therefore leak subtraction was not used. The average cell capacitance was ∼10 pF. Series resistance was compensated between protocols to 70% (prediction and correction; 10 µs lag), resulting in maximal residual voltage error below 1.6 mV during measurement of the current-voltage (*I-V*) relationship. All experiments were performed at room temperature. Access resistance and cell capacitance were measured using on-line exponential fits to a capacitance transient (Membrane Test, Clampex). Access resistance averaged ∼4 MΩ. Data from cells where the access resistance exceeded 5.5 MΩ were discarded. Activation and inactivation kinetics were calculated simultaneously using double exponential fits to the current trace using Clampfit 9.0 software (Axon Instruments). Steady-state activation and inactivation curves from each cell were fit in Excel using the Solver function, then averaged. Average results are reported in Table along with the s.e.m and number of cells.

We used the method of Agler et al., to estimate the probability of channel opening, *P_o_* [19], which assumes no change in single channel current, reducing the relationship between whole cell current (*I*) to *I ≈ NP_o_*, where *N* is the number of channels in a cell and *P_o_* is the probability of channel opening. *N* is estimated by measuring the channel gating current at the reversal potential for ionic current [19]. The area under this current represents the maximal gating charge *Q*_max_, and is proportional to *N*. As described previously [4], peak ionic current conductance, *G*_max_, was determined by fitting the *I-V* curve, obtained from the same cell, with a Boltzmann-Ohm equation. *G*_max_ is used as a proxy for *I* since it is not affected by changes in driving force, and some mutants open at more negative potentials where the driving force for Ca^2+^ entry is larger than control. Therefore, the G_max_/Q_max_ ratio can be used to estimate *P_o_* [19].

### Luminometry

HEK-293 cells were cultured in 24-well plates and transfected using JetPEI (Ozyme, France) with 0.5 µg of DNA per well of the various HA-tagged mutants of Ca_V_3.1, Ca_V_3.2, and Ca_V_3.3 [18]. The luminometric measurements were performed 48 h after transfection. Briefly, cells were rinsed and fixed for 5 min in 4% paraformaldehyde and then washed three times for 5 min with PBS. Half of the wells were permeabilized with 0.1% Triton X-100 for 5 min and rinsed three times with PBS. Cells were then incubated for 30 min in blocking solution (PBS plus 1% FBS). The HA–Ca_V_3 proteins were detected using a monoclonal rat anti-HA antibody (1∶1000, clone 3F10; Roche Diagnostics) after incubation for 1 hour at room temperature. After extensive washes (four times for 10 min in PBS plus 1% FBS), cells were incubated for 30 min with the secondary goat anti-rat antibody coupled to horseradish peroxidase (1∶1000; Jackson ImmunoResearch, West Grove, PA). Cells were rinsed four times for 10 min with PBS before addition of SuperSignal enzyme-linked immunosorbent assay femto maximum sensitivity substrate (Pierce, Rockford, IL). The luminescence was measured using a Victor 2 luminometer (PerkinElmer, Wellesley, MA), and then protein amount in each well was measured with a BCA assay (Pierce) to normalize the measurements. The data were also normalized to the level of signal obtained for the wild-type (WT) HA–Ca_V_3 protein in the non-permeabilized condition. Seven to 12 independent sets of transfections were performed for each condition. Results are presented as mean±s.e.m., and statistical differences were evaluated using Student's t-test.

## Results

### Rationale for deletions

Previous studies on Ca_v_3.2 have shown that the proximal portion of the I–II loop contains a gating brake that restrains channels from opening, while the distal I–II loop contains determinants that regulate channel trafficking to the plasma membrane [3], [4]. In a recent study we mapped the end of the gating brake in Ca_v_3.2 to the end of a helix-loop-helix structure [4]. In keeping with our initial deletion naming scheme [3], we call deletions of the brake region D1–2 (Fig. 1), and for simplicity refer to the channels by their original α1X nomenclature, such that deletions in Ca_v_3.1 (α1G) are termed GD1–2 and Ca_v_3.3 (α1I) are termed ID1–2. Likewise, deletions D3–5 (D3, D4, and D5) removed the distal portion of the I–II loop, sparing the D6 region that was found to affect channel gating in Ca_v_3.2 [3].

### D1–2 and D3–5 deletion mutants reveal a hyperpolarized shift in the voltage dependence of activation vs. wild-type Ca_v_3s

**Figure 2:**
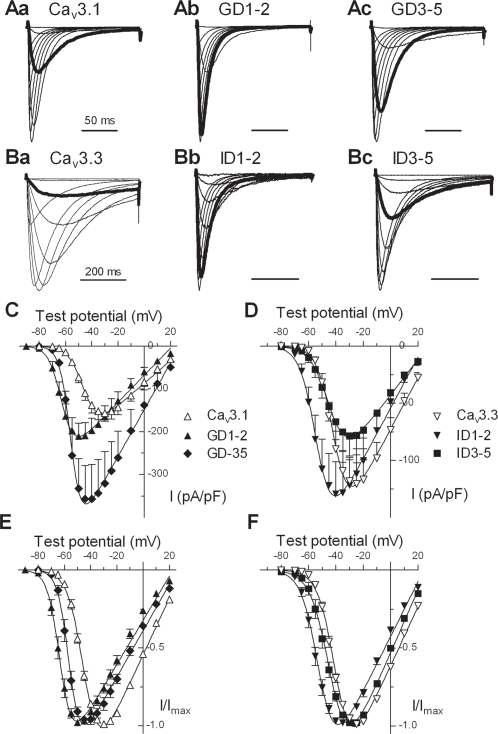
Effect of Ca_v_3.1 I–II loop deletions on the voltage dependence of channel activation. *A, B,* Normalized current traces recorded during depolarizing voltage steps from −80 to +30 mV (holding potential, −100 mV, except for GD1–2 mutant, which due to shifted inactivation was −110 mV) in WT Ca_v_3.1 (*Aa*), GD1–2 (*Ab*), GD3–5 (*Ac*), WT Ca_v_3.3 (*Ba*), ID1–2 (*Bb*) and ID3–5 (*Bc*). Thick gray lines represent the current at −50 mV, demonstrating the negative shift in voltage dependence of activation observed in the deletion mutants. Currents were normalized to the maximum peak current in that cell. Time calibration bar scale applies to all three sets of traces in each case. Peak current-voltage plots for either Ca_v_3.1 and its deletions (*C*) or Ca_v_3.3 and its deletions (*D*). Peak currents were normalized to the cell size as estimated by capacitance. Normalized current-voltage plots for either Ca_v_3.1 and its deletions (*E*) or Ca_v_3.3 and its deletions (*F*). Same symbol definition as in panels *C* and *D*. Smooth curves in *C–F* represent fits to the average data using a Boltzmann–Ohm equation [4].

To determine the effects of the initial region of the I–II loop on channel gating, we transfected HEK-293 cells with the deletion mutants of Ca_v_3.1 and Ca_v_3.3. The first deletion, D1–2, removes highly conserved amino acids in the I–II loop that are immediately proximal to the transmembrane segment of the first repeat (IS6; Fig. 1). The voltage-dependent of activation of D1–2 channels was significantly shifted to more hyperpolarized potentials (Fig. 2). This shift can can be appreciated in both the current density and normalized *I-V* plots, and reflect a shift caused by the GD1–2 and ID1–2 deletions of −18 and −11 mV, respectively (Fig. 2, Table 1). The deletion mutants of Ca_v_3.1 were more sensitive to voltage changes, as evidenced by a decrease in the slope factors that describe the activation curves (Table 1). The deletion mutants are distinguishable by their variable effects on gating, with D1–2 generally exerting more profound effects compared with D3–5 (Fig. 2; Table 1).

**Table 1:** Electrophysiological properties of Ca_v_3.1, Ca_v_3.3, and their deletion mutants.

Current Density			Activation					Inactivation					P_o_ estimate		
	*G_max_*	sem	*V_0.5_*	sem	*k*	sem	n	*V_0.5_*	sem	*k*	sem	n	*G/Q*	sem	n
	(nS/pF)		(mV)		(mV)			(mV)		(mV)					
Ca_v_3.1 WT	2.9	±0.3	−44.6	±0.7	5.8	±0.2	19	−77.7	±0.7	−5.0	±0.2	15	0.33	±0.05	9
GD1–2	4.5	±0.8^*^	−62.2	±5.8^**^	4.6	±0.2^***^	9	−91.0	±1.6^***^	−4.0	±0.1^***^	9	0.94	±0.19^**^	6
GD3–5	6.0	±1.2^**^	−56.0	±1.2^***^	4.0	±0.3^***^	8	−81.2	±1.5^*^	−4.2	±0.2^*^	8	0.31	±0.03	7
Ca_v_3.3 WT	2.3	±0.3	−40.2	±0.8	5.8	±0.2	17	−68.6	±0.7	−5.5	±0.5	9	0.16	±0.02	9
ID1–2	2.4	±0.5	−51.6	±1.4^***^	5.9	±0.2	9	−83.0	±1.1^***^	−4.3	±0.2	6	0.32	±0.04^***^	6
ID3–5	1.6	±0.3	−43.2	±1.5	6.6	±0.2^*^	8	−76.1	±1.7^***^	−5.1	±0.2	5	0.17	±0.01	6

The *G_max_* and *V_0.5_* of activation were determined from the *I-V* protocol, and therefore have the same number of cells (n) in each measurement. The *G/Q* ratio was calculated for each individual cell, and then averaged. Statistical significance is denoted with asterisks, where three asterisks indicates P<0.001, two for P<0.01, and one for P<0.05.

### D1–2 deletion mutants display a hyperpolarized shift in steady-state inactivation vs. wild-type Ca_v_3s

**Figure 3:**
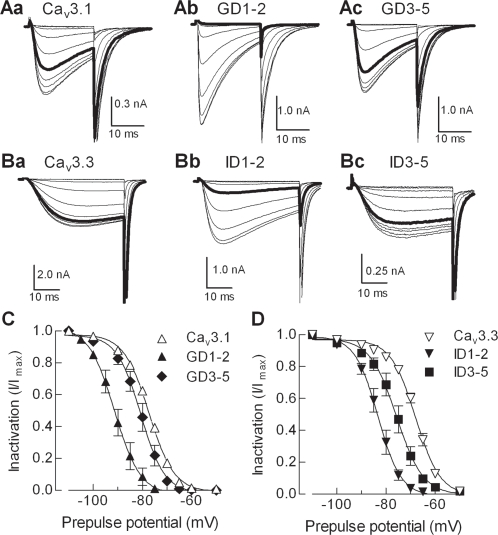
Effect of deletions on steady-state inactivation. *A, B*, Representative current traces obtained during the 20 ms test pulse to −20 mV to measure channel availability. This test pulse was preceded by incremental hyperpolarizing pulses (15 s) from −110 mV in WT Ca_v_3.1 (*Aa*), GD1–2 (*Ab*), GD3–5 (*Ac*), and WT Ca_v_3.3 (*Ba*), ID1–2 (*Bb*), ID3–5 (*Bc*). Thick gray lines represent the current available after prepulses to −80 mV, demonstrating the negative shift in steady-state inactivation in the deletion mutants. *C, D,* The mean normalized amplitude of the current is expressed as a function of prepulse potential and fit with a Boltzmann equation (smooth curves). Averages of the fits to data from individual cells are reported in Table 1.

We next examined contributions of the D1–2 region of the I–II loop of Ca_v_3.3 to steady-state inactivation (h_∞_). The voltage dependence of channel inactivation was measured using prepulses to varying potentials for 15 s and a 50 ms test pulse to −35 mV to determine channel availability. In agreement with previous findings with Ca_v_3.2 [3], deletion of the gating brake in both Ca_v_3.1 and Ca_v_3.3 produced a hyperpolarized shift in the steady-state inactivation curves (Fig. 3). Curiously, despite the large shift in the voltage-dependence of activation, the GD3–5 deletion only had a small −3 mV shift of the steady-state inactivation curve (Fig. 3C, D, Table 1). In contrast, the ID3–5 deletion shifted the inactivation curve (8 mV) more than the activation curve (3 mV). These results are in line with our prediction that the D1–2 region of the I–II loop serves as a conserved gating brake in all LVA Ca^2+^ channels, but begins to reveal differences in the role of the I–II loop in gating of Ca_v_3.3.

### D1–2 deletions alter activation and inactivation kinetics

**Figure 4:**
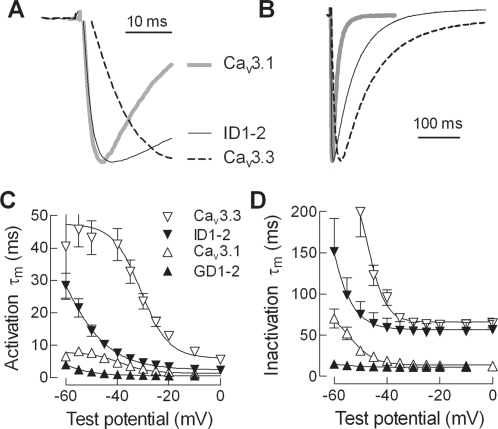
Effect of D1–2 deletions on kinetics of Ca_v_3.1 and Ca_v_3.3 kinetics. *A*, *B,* Normalized current traces for Ca_v_3.1 (thick gray line), Ca_v_3.3 (dashed line), and ID1–2. Currents were recorded during step depolarizations to −10 mV. The same current traces are shown in *A* and *B*, but at a different time scale. In *A* the time scale is expanded to illustrate how ID1–2 activates as fast as Ca_v_3.1, while in *B* a longer time scale is shown to illustrate how ID1–2 inactivates at a similar rate as WT Ca_v_3.3. *C,* Average activation kinetics estimated using a 2 exponential fit to the raw current traces obtained during the *I-V* protocol. Data represent mean±s.e.m , and N is shown in Table 1. *D,* Average inactivation kinetics. Same symbol definition as in panel *C*.

A distinguishing feature of Ca_v_3.3 channels is their slow activation and inactivation kinetics, which allows them to contribute to long-lasting bursts of firing as in neurons of the reticular thalamic nucleus (nRT) [7], [8]. Despite extensive chimeric analysis, a structural region responsible for these kinetic differences has not been identified [10], [20]. A hallmark of any mutation that disrupts the gating brake of Ca_v_3.2 channels is accelerated kinetics of both activation and inactivation [3], [4]. Therefore, we analyzed the kinetics of the mutant channels by fitting the current traces obtained during the *I-V* protocol with two exponentials, one to estimate activation kinetics and the second to estimate the apparent rate of inactivation. As expected, the D1–2 deletion in Ca_v_3.1 accelerated both activation and inactivation kinetics (Fig. 4). Importantly, the D1–2 deletion in Ca_v_3.3 created channels that activated almost as fast as WT Ca_v_3.1 channels (Fig. 4A,C). T-channel kinetics are slow at near threshold potentials (−50 mV), and accelerate to a voltage-independent rate near −10 mV. Again, ID1–2 channels resembled WT Ca_v_3.1 channels, showing less voltage-dependence in activation kinetics (Fig. 4C). In sharp contrast, inactivation kinetics were only accelerated at negative potentials (<−40 mV), with no difference between ID1–2 and WT Ca_v_3.3 channels at more positive potentials (Fig. 4D). These results clearly show that the gating brake is not only involved in setting the voltage sensitivity of T-channels, but also in determining their kinetics.

### Effects of the I–II loop deletion mutants of Ca_v_3.1 and Ca_v_3.3 on surface expression

**Figure 5:**
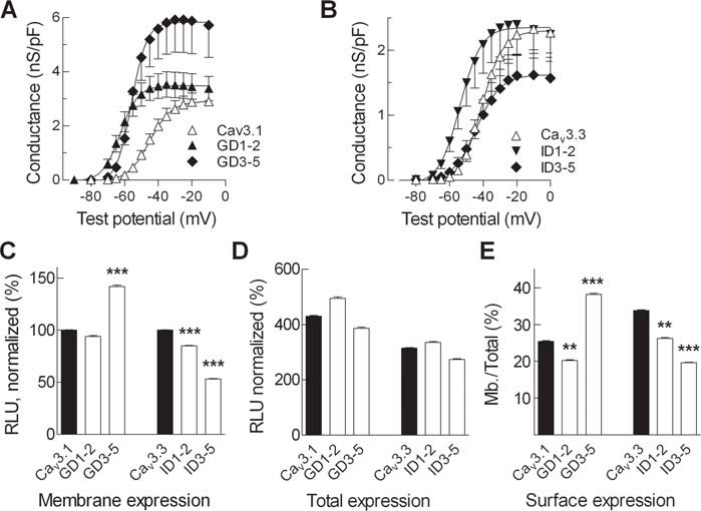
Effect of deletions on channel surface expression as measured by electrophysiology and luminometry. *A,B,* The *I-V* data from each cell was fit with a Boltzmann-Ohm equation to calculate *V_0.5_*, *k*, *G_max_*, and *E_rev_*. The value of *E_rev_* was then used to calculate the chord conductance at each test potential. These data were then normalized for cell capacitance. Only the increase in GD3–5 conductance (G_max_) was statistically different than control Ca_v_3.1. *C,* Luminometric quantification of the expression levels of HA-tagged Ca_v_3.1 and Ca_v_3.3 channel variants at the membrane (non-permeabilized). Average relative light units (RLU) for WT channels before normalization were: Ca_v_3.1, 2,867,417±551,473; and Ca_v_3.2 2,533,150±353,578, n = 6 for both). To reduce the error between experiments the data were normalized to the membrane expression for the respective WT channel. Total expression of HA-tagged channels measured after Triton X-100 permeabilization normalized to membrane expression. Ratio of surface/total expression identifies an increase in membrane expression for GD3–5, and decreases in GD1–2, ID1–2, and ID3–5. Statistically significant differences from control wild-type channels are indicated (**p<0.01; ***p<0.001).

The currents recorded from cells transiently transfected with GD1–2 and GD3–5 channels were significantly larger than WT Ca_v_3.1 (Fig. 2C). Since these channels open at more hyperpolarized potentials, we calculated the apparent maximal conductance (G*_max_*) to account for the increased driving force for Ca^2+^ entry, and adjusted for cell size (Fig. 5A). This analysis revealed that the increase in GD1–2 channels was solely due to changes in driving force, as channels opened at potentials (−60 mV) further away from the apparent reversal potential (+40 mV). In contrast, *G_max_* for GD3–5 currents was 2-fold higher than WT Ca_v_3.1 (Fig. 5A, Table 1). In contrast, the ID1–2 and ID3–5 mutations had no significant effect on current density (Fig. 5B, Table 1).

Luminometry was used to analyze cells expressing a surface accessible HA epitope, thereby providing an independent method to concomitantly measure the percentage of cells expressing the channel at the surface and the level of surface expression. The I–II loop deletions were introduced into a modified human Ca_v_3.1 or Ca_v_3.3 channel construct containing an extracellular HA (located in the IS5-pore loop) tag to measure their surface (non-permeabilized; Fig 5C) and total expression (permeabilized, Fig. 5D). The ratio of these two signals provides a measure of the fraction of channels at the plasma membrane. Previous studies have shown that this measure correlates with surface expression as assessed by either FACS or confocal microcopy [3]. The first notable finding was that Ca_v_3.1 and Ca_v_3.3 channels trafficked more efficiently to the membrane than Ca_v_3.2 channels, showing 25.4% and 33.8%, respectively, of the total channel pool was localized at the membrane (membrane/total; Fig. 5E), in contrast to only 12.5% of Ca_v_3.2 channels localized at the membrane [3].

In contrast to our previous findings with D3–5 deletions in Ca_v_3.2, which increased surface expression to 4-fold [3], the similar D3–5 deletion in Ca_v_3.1 only increased surface expression from 25.4 to 38.2%. Unexpectedly, the analogous D3–5 deletion in Ca_v_3.3 decreased expression from 33.8 to 19.6% (Fig. 5E). Similarly, the D1–2 deletion in both Ca_v_3.1 and Ca_v_3.3 slightly decreased surface expression, whereas in the previous study it had increased Ca_v_3.2 expression. These results clearly demonstrate that the role of the I–II loop in trafficking channels is not conserved across all Ca_v_3 channels. Deletions in the I–II loop increase the surface expression of Ca_v_3.1 and Ca_v_3.2 channels, suggesting that the dominant role of this loop is to prevent surface expression. In contrast, the present results suggest that the loop in Ca_v_3.3 channels plays an opposite role by aiding the trafficking of channels to the plasma membrane, as deletion of the distal region of the I–II loop of Ca_v_3.3 produced diminished surface expression (Fig. 5C).

### Effects of the I–II loop deletion mutants of Ca_v_3.1 and Ca_v_3.3 on P_o_

**Figure 6:**
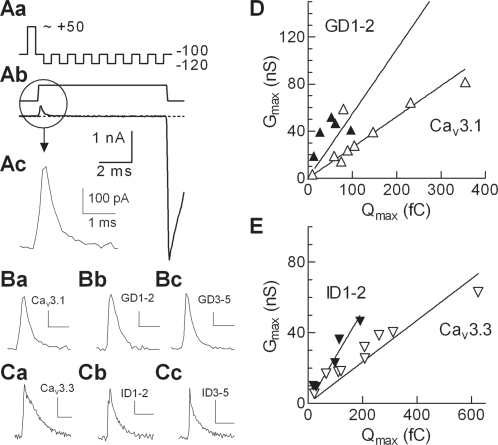
Estimating the effect of the deletions on the probability of channel opening. *Aa*, Schematic of the P/-8 voltage protocol. *Ab*, Representative current at full scale, which is expanded in panel *Ac*. *B,C* Representative gating current traces recorded during depolarizing voltage steps from −100 to ∼ +50 mV (reversal potential): WT Ca_v_3.1 (*Ba*); GD1–2 (*Bb*); GD3–5 (*Bc*); WT Ca_v_3.3 (*Ca*); ID1–2 (*Cb*); and ID3–5 (*Cc*). Vertical scale bar is same size for all six traces (0.1 nA), while the horizontal scale bar is 1 ms in *B* and 2 ms in *C*. Data were acquired at 20 kHz, filtered at 10 kHz, and represent the average of 20 runs. G_max_ vs. Q_max_ for WT Ca_v_3.1 and GD1–2 (*D*), or WT Ca_v_3.3 and ID1–2 (*E*). The slope of the linear regression fit provides an estimate of *P_o_,* and in both cases the slope of the line fitting the D1–2 mutants was 2-fold higher than for WT (Ca_v_3.1, 0.26±0.03, n = 9; GD1–2, 0.55±0.06, n = 6, P<0.001; Ca_v_3.3, 0.12±0.01, n = 9; and ID1–2, 0.26±0.02, n = 6, P<0.05). The difference between Ca_v_3.1 and Ca_v_3.3 is also statistically significant (P<0.001, one-way ANOVA followed by Tukey's multiple comparison test, Prism).

We next investigated whether changes in the probability of channels opening contributed to the effects on maximal conductance. We assayed surface expression by measurement of the channel gating current, which consists of charge movements within the positively charged S4 transmembrane segments and provides a linear approximation of the number of channels localized to the plasma membrane [21]. Evidence suggests that depolarization of the membrane potential induces conformational changes in this segment that, in turn, lead to opening of the channel pore. Specifically, depolarization causes the S4 segment to extend towards the outer phase of the lipid bilayer. Gating currents are measured after negating ionic current by depolarizing the cell to the observed reversal potential. The transient current observed includes gating current along with capacitive and leak currents. These confounding sources of current were subtracted from the transient, leaving only gating current (P/-8 protocol; Fig. 6A). Relatively large gating currents (average 0.4 nA) were observed with all constructs tested (Fig. 6 Ba-Cc). As detailed in the Methods, the *G_max_* vs. *Q_max_* ratio provides an estimate of channel *P_o_* [19], and this can be appreciated by the either slope of the line (Fig. 6D,E) or by averaging the G/Q ratio from individual cells (Table 1). Notably, the estimated *P_o_* of Ca_v_3.1 channels was 2-fold higher than for Ca_v_3.3 channels using either method.

Deletion of the gating brake produced a 2-fold increase in the estimated *P_o_* in both Ca_v_3.3 (ID1–2) channels and Ca_v_3.1 (GD1–2) channels (Fig. 6D,E, Table 1). In neither channel did the D3–5 deletion affect this estimate of channel *P_o_*, suggesting that the increase in current density with the Ca_v_3.1 D3–5 mutant was due to changes in the number of channels at the plasma membrane (Table 1).

## Discussion

The present study identifies that structural alterations into the I–II loop of Ca_v_3 channels yield large effects on T-channel activity and demonstrates significant differences among the Ca_v_3 subtypes in the ability of this intracellular domain to direct expression at the plasma membrane of T-channels. Considering the present data obtained for Ca_v_3.1 and Ca_v_3.3, together with recent results regarding Ca_v_3.2 [3], [4], we are able to provide the first comprehensive molecular framework for how the I–II loop regulates activity of the three Ca_v_3 channels. Notably, the three Ca_v_3 subunits have in common a proximal domain of the I–II linker that contributes to the gating properties. In contrast, only in the Ca_v_3.2 subunit does the central region of the I–II loop play a critical role in the trafficking to the plasma membrane, as we only observed modest effects of loop deletions on the surface expression for both Ca_v_3.1 and Ca_v_3.3 channels. The I–II loop of Ca_v_3 channels appears to be a major locus for T-channel modulation: a checkpoint for both trafficking and gating properties, reminiscent to that described for HVA channels.

### The role of proximal I–II loop in Ca_v_3 gating

The very proximal I–II loop is a highly conserved structural determinant in all Ca_v_3 channels. Its involvement in T-channel gating has been identified in our recent study on Ca_v_3.2 [3] and further substantiated by a detailed structure-function analysis of this domain [4]. Secondary structure prediction programs suggest this region may form a helix-loop-helix structure, where helix extends the brake region away from IS6, and the second helix returns back towards the channel. This model was supported by mutagenesis studies where the helices were either disrupted by replacing them with poly-proline-glycine, or by maintaining them by replacement with poly-alanines [4]. Disruption of the helix-loop-helix region in the Ca_v_3.2 channel produced robust negative shifts in activation and inactivation properties, as well as faster kinetics [3], [4]. Similarly, we report here that deletion of this D1–2 proximal region in both Ca_v_3.1 and Ca_v_3.3 channels yields large and highly significant alterations in activation and inactivation properties (see Table 1), which fully mimics removal of the gating brake locus in Ca_v_3.2 [4]. Overall, the data obtained on D1–2-deleted Ca_v_3 channels reveal that this domain critically contributes to T-channel gating in all three subtypes.

Deletion of the more central region of the I–II loop (D3–5) also yields moderate but significant changes in Ca_v_3.1 and Ca_v_3.3 channel gating. A similar result was obtained with the D3–5 deletion in Ca_v_3.2 [3]. Oddly, effects on gating were not observed in D3, D4, and D5 deletions of Ca_v_3.2. Two interpretations of this result are: one, that the proximal D1–2 region represents the active component of the I–II loop gating locus, while the adjacent D3–5 domain only partially contributes to it as a modulatory component; or two, that linking the brake directly to the pre-IIS1 region introduces such a conformational strain to partially disrupt its function. Since the D1–2 region is highly conserved in Ca_v_3 channels, we believe the deletion strategy we have developed was instrumental in pinpointing the respective roles of the D1–2 and D3–5 regions in T-channel activity. Several previous studies using a chimera strategy have not conclusively succeeded in identifying the molecular determinants in Ca_v_3 channels supporting the specific T-channel gating properties. Park et al. [10] and Hamid et al. [11] concluded that multiple channel structural determinants appear to control kinetics and inactivation, respectively, of Ca_v_3 channels. Interestingly, other evidence, using both experimental and modeling approaches, suggests that intracellular components of the pore, most likely the S6 segments, also contribute to both the activation and inactivation gates of T-type channels [22]. Our results show that the structural integrity of the brake structure is critical for the normal low voltage-activation of this T-type channel. Deletion of this region leads to channels that open at more negative potentials and inactivate at more negative potentials. As noted previously, inactivation of T-channels is coupled to activation [22], which may explain the concomitant shifts in activation and inactivation kinetics observed with GD1–2. In contrast, deletion of the brake in Ca_v_3.3 accelerated activation kinetics with little effect on inactivation kinetics. This result suggests the brake is a major determinant of the distinctive slow activation kinetics of Ca_v_3.3 channels. The conclusion from chimeric studies is that a number of regions contribute to Ca_v_3.3 inactivation kinetics, and our results exclude the gating brake. Previous measurements of T-channel gating currents indicate that 80% of the channels open after only 20% of total charge movement [23], [24], which implies that T-channels open after minimal movement of their voltage sensors, and modeling studies suggest this could explain why T-channel kinetics are so voltage-dependent [25]. We propose that the brake region stabilizes the closed state, adopting the role of one of the S4–S5 linkers to keep the pore closed at resting membrane potentials [4]. In this scheme, disruption of the brake allows channels to open at more negative membrane potentials, and open faster. Finally, helix 2 of the brake region in Ca_v_3.3 is much less conserved among Ca_v_3 channels than helix 1, suggesting interesting differences in the role of the brake in setting the activation kinetics. Overall, our study, which provides novel insights into the molecular gating domains of T-channels, should provoke more detailed structure-functional analysis to investigate how these adjacent domains, which include the S4–S5 linker, S6 segment as well as proximal and central regions of the I–II loop, cooperate to set up T-channel gating.

### The role of the central I–II loop in Ca_v_3 surface expression

Based on the data collected on the Ca_v_3.2 channel, it was tempting to hypothesize that the I–II loop modulates cell surface trafficking of all Ca_v_3 channels [3]. Our present study was therefore designed to investigate this property in Ca_v_3.1 and Ca_v_3.3 channels. For this purpose, HA-tagged constructs were developed, verified for their ability to produce functional T-channels, and assayed in luminometry experiments. A first notable result was that both Ca_v_3.1 and Ca_v_3.3 were expressed significantly more at the plasma membrane than for Ca_v_3.2 [3]. Importantly, both the patch-clamp data (conductance) and luminometry data (membrane/total ratio) were in agreement in revealing that a D3–5 deletion in the Ca_v_3.1 induces a modest ∼2-fold increase in plasma membrane expression. At first glance, this result was unexpected as this D3–5 deletion in the Ca_v_3.2 channel was shown to result in a 6.7-fold increase in currents and 3.7-fold increase in surface expression [3]. Altogether, these data indicate that the D3–5 region in Ca_v_3.2 harbors unique properties in terms of modulating channel trafficking towards the plasma membrane. The opposite behavior can be deduced from our findings: wild-type Ca_v_3.1 and Ca_v_3.3 channels show significantly higher expression at the plasma membrane in comparison to Ca_v_3.2, while deletion of a central I–II loop domain (D3–5) favors surface expression mainly for Ca_v_3.2 channels. Although the D1–2 domain is highly conserved among Ca_v_3 channels, that is not the case for the D3–5 region (Figure 1). We therefore propose that a molecular determinant contained in Ca_v_3.2, but absent in Ca_v_3.1, which is closer in terms of sequence homology, plays a major role in targeting Ca_v_3.2 to the plasma membrane. A thorough structure-function analysis of this region should help to identify the amino-acids involved in this process. To date, little is known about the important determinants within the I–II loop of Ca_v_3.2 that are involved in surface expression. We previously reported that several domains may cooperate in this process, as sequential deletions within the I–II loop of Ca_v_3.2 resulted in a significant increase in surface expression of the protein, with deletion of the D3–5 region yielding the strongest effects [3].

### Physiological and pathophysiological implications

To date, the only known SNP/mutation in the I–II loop of Ca_v_3.1 has been found in a patient exhibiting a sporadic case of juvenile myoclonic epilepsy (JME) with early childhood absence and astatic seizures [26]. This point mutation (A570V), which is localized in the D3–5 domain, produces no change in T-current density. These data are in good agreement with our present observations describing molecular alterations in the I–II loop that result in minor changes in surface Ca_v_3.1 expression and current density. To our current knowledge, no mutation in the I–II loop of Ca_v_3.3 has been reported to date. In contrast, many SNPs/mutations were found in the Ca_v_3.2 channel of patients with childhood absence epilepsy (CAE) in a Chinese population [5], [27]. Notably, many of these mutations localize within the I–II loop, which can be considered a hot-spot of CAE mutations. Of interest, surface expression of Ca_v_3.2 channels harboring single amino-acid mutations within the I–II loop was significantly increased [3], further validating our structure-function analysis of the I–II loop of Ca_v_3 channels. Whether splice variations occurs in the I–II loop of Ca_v_3 channels is another important consideration. To date, only splice variation of human Ca_v_3.3 genes has been reported [28], [29], and the effects on channel trafficking were not explored.

Studies have documented both divergent biophysical properties and physiological roles for the three Ca_v_3 channels of T-channels. Indeed, electrophysiological analysis of the cloned Ca_v_3.3 channel demonstrated that it was an LVA channel but with distinct kinetic properties in comparison to Ca_v_3.1 and Ca_v_3.2 [30], suggesting a unique role for each Ca_v_3 channel in neuronal excitability. Several lines of evidence suggest that the Ca_v_3.2 channel is subject to a wide variety of modulations capable of dynamically fine-tuning channel activity in neurons. Indeed, the Ca_v_3.2 channel is selectively modulated by several endogenous ligands as zinc, reducing agents, and ascorbate [31]–[33], as well as G protein βγ proteins [34], [35]. Interestingly, it was also reported that the density of T-current supported by Ca_v_3.2 channel can vary in neurons in several disease states, such as temporal lobe epilepsy [36] or diabetic neuropathy [37], further indicating that surface expression of Ca_v_3.2 channels is a critical index of its functional expression. By showing that the I–II loop is a major checkpoint for surface expression only for the Ca_v_3.2 channel, our data open new insights into the multimodal modulation pathways of this T-channel subspecies. It is interesting to note that the I–II loop of HVA channels also plays a prominent role in the gating and expression of these channels, although in this case Ca_v_β subunits play a key role in both trafficking channels to the surface and increasing channel P_o_ [6]. Although Ca_v_β subunits can alter the surface expression of LVA channels [18], further work is required to establish whether native LVA channels associate with these auxiliary subunits [38], [39], or novel proteins.

Characterizing the structure and function of the low voltage-activated Ca_v_3.1 and Ca_v_3.3 channels may clarify how aberrant mutations underlie channel dysfunction. This leads to the hope that novel genetic and pharmacological therapies that target voltage-gated Ca^2+^ channels will treat these insidious mental diseases more efficaciously. The studies here aim to illuminate the structure and function of voltage-gated Ca^2+^ channels, which can not only serve to refine the molecular targets of existing drugs, but also to uncover targets for future pharmaceutical therapies. Furthermore, these findings have the potential to enhance our understanding of the cellular and molecular mechanisms underlying the regulation of low voltage-activated Ca^2+^ channels, as well as provide novel structural insights that can be used to treat seizures and various other pathological phenomena. Most significantly, these experiments help to establish a unified property of the proximal region of the I–II loop in LVA channels.
